# MXene-Reinforced Composite Cryogel Scaffold for Neural Tissue Repair

**DOI:** 10.3390/molecules30030479

**Published:** 2025-01-22

**Authors:** Mohamed Zoughaib, Svetlana Avdokushina, Irina N. Savina

**Affiliations:** 1 Institute of Fundamental Medicine and Biology, Kazan (Volga Region) Federal University, 18 Kremlyovskaya St., 420008 Kazan, Russia; mohamadzougheib@gmail.com (M.Z.); sveta.avdokushina@gmail.com (S.A.); 2 Scientific and Educational Center of Pharmaceutics, Kazan (Volga Region) Federal University, 18 Kremlyovskaya St., 420008 Kazan, Russia; 3 School of Applied Sciences, University of Brighton, Huxley Building, Lewes Road, Brighton BN2 4GJ, UK

**Keywords:** MXene, cryogel, neural cells, neuroregeneration, neuroprotective properties, spinal cord injury

## Abstract

The development of effective materials for neural tissue repair remains a major challenge in regenerative medicine. In this study, we present a novel MXene-reinforced composite cryogel scaffold designed for neural tissue regeneration. MXenes, a class of two-dimensional materials with high conductivity and biocompatibility, were integrated into a polyvinyl alcohol (PVA) matrix via cryopolymerization to form a macroporous, mechanically stable scaffold. The morphology, mechanical properties, and swelling behavior of the cryogel with different MXene contents have been assessed. The effects of MXene on the viability/proliferation and differentiation of neural cells (PC-12) cultured in the composite cryogel were elucidated. The MXene/PVA cryogel demonstrated excellent cell-supporting potential, with MXene not only showing no toxicity but also promoting the proliferation of cultured PC-12. Additionally, MXene induced a neuritogenesis-like process in the cells as evidenced by morphological changes and the enhanced expression of the neural marker β-III-tubulin. The neuroprotective properties of the MXene component were revealed by the alleviation of oxidative stress and reduction of intracellular ROS levels. These findings highlight the potential of MXene-embedded PVA cryogel as a promising material that can be further used in conjunction with electrostimulation therapy for advancing strategies in neural tissue engineering.

## 1. Introduction

Traumatic injuries to the spinal cord and peripheral nervous system represent a major medical problem that causes significant burden on healthcare systems and profoundly diminished quality of life for affected individuals worldwide [1]. Nerve repair remains a great challenge for researchers and clinicians, where the poor availability of donor tissues, size mismatch, induction of immune response, and morbidity of the donor site are the main limitations in clinical use of autologous and allogeneic grafts [2,3,4].

Biomaterials have emerged as promising alternatives that overcome the drawbacks of standard treatments [5,6]. The use of polymeric scaffold implants as bridging biomaterials helps to reduce scar and cyst formation and imitates the extracellular matrix (ECM) functions by providing spatial continuity across the defect and supporting local cell migration and survival [7]. A successful scaffold should be endowed with appropriate chemical and physical properties as well as electrical conductivity. Conductive scaffolds are suitable candidates for the development of nerve conduits due to their ability to enhance neurite outgrowth, promote axonal regeneration, and improve neural differentiation compared with non-conductive scaffolds, especially when combined with external electrical stimulation [8,9,10,11]. An attractive advantage offered by conductive substrates is the modulation of neural cell behavior without altering the genome using exogenous genetic material or chemical stimuli [12].

Electroconductive materials can be obtained by blending their polymer constituents with conductive particles such as graphene and carbon nanotubes [13,14]. However, their use in biomedical applications is restricted due to their cytotoxicity [15,16]. A promising substitute is provided by MXenes, a family of two-dimensional transition metal carbides/nitrides that, in recent years since their discovery, have attracted significant attention due to their low toxicity, excellent electroconductivity, and cost-effectiveness [17]. The abundance of hydrophilic functional groups (OH, O, and F) exposed on MXene exteriors contributes to their hydrophilicity, facilitates surface modification, and improves their biocompatibility, whereas the presence in their composition of relatively inert metals combined with carbonitrides supports their cell-friendly conduct [17,18]. MXenes have been used in biosensing [19], antibacterial and antiviral materials [20,21], theranostics [22], sensors [23], and tissue engineering [24,25].

Owing to the desirable characteristics and proven biocompatibility of 2D MXene nanosheets, particularly towards neural stem cells (NSCs) [26,27,28], several recent studies have highlighted the potential of using MXenes in combination with hydrogels to create conductive 3D composite materials that simulate the ECM for neural tissue repair. For instance, Ti_3_C_2_T_x_ MXene–Matrigel hydrogels have been shown to promote the proliferation and differentiation of NSCs [29] and neurite outgrowth of spiral ganglion neurons [30]. The polyvinylpyrrolidone/phytic acid/MXenes hydrogel demonstrated good biocompatibility in vitro with mouse neuroectodermal stem cells and significantly accelerated angiogenesis, remyelination, and axon regeneration after injection into a rat model of complete spinal cord injury (SCI) [31]. Likewise, gelatin methacrylate hydrogels modified with MXene promoted NSC proliferation and differentiation in vitro and led to a remarkable recovery of hind limb motor function, as evidenced by increased BBB scores in rats with SCI [32].

The MXene-incorporating scaffolds investigated to date still have certain shortcomings, in particular the lack of sufficient porosity, which is recompensed by the introduction of microstructures such as grooves through laborious techniques [32]. In addition, most of these materials consist of natural polymers and their derivatives which, despite their biocompatibility and partially preserved ECM functionalities, do not provide precise control over the biochemical and structural properties of the developed materials and suffer from inherent immunogenicity [33]. Developing these hydrogels using cryotropic gelation is a significant step toward producing macroporous synthetic analogs, known as cryogels. These cryogels are characterized by a unique interpenetrating porous structure supporting gas and nutrient exchange, as well as cell infiltration and growth, in a three-dimensional environment with increased availability for biointeractions [34].

Herein, we describe, for the first time, the development of a MXene-embedded polyvinyl alcohol (PVA) cryogel as a composite biomaterial for potential application in nerve repair. The effects of the introduced MXene content on the formation of the polymer network, modulation of the mechanical properties of macroporous cryogels, neural cell viability within the matrix, and ROS scavenging were elucidated. The MXene content was optimized to support neural cell proliferation and differentiation, and analyzed in terms of its antioxidant abilities. The resulting MXene-functionalized PVA cryogel can be considered a platform for the development of biomaterial candidates for neural tissue replacement.

## 2. Results and Discussion

### 2.1. Preparation and Characterization of MXene/PVA Cryogels

Ti_3_C_2_T_x_ MXenes, obtained by etching the Al layer from MAX-phase Ti_3_AlC_2_ [35,36,37], were incorporated into the cryogel during their preparation as detailed in Section 3. PVA cryogels with different concentrations of MXenes were obtained using the cryotropic gelation technique [38,39]. The choice of the MXene concentration range (100–500 µg/mL) was based on previous studies where these particles were combined with different types of hydrogels intended for neural tissue engineering [29,32]. The final concentration of MXene in the PVA solution was 0, 100, 200, or 500 μg/mL. The samples were labeled as PVA-M_0_, PVA-M_100_, PVA-M_200_, and PVA-M_500_, respectively.

**Figure 1:**
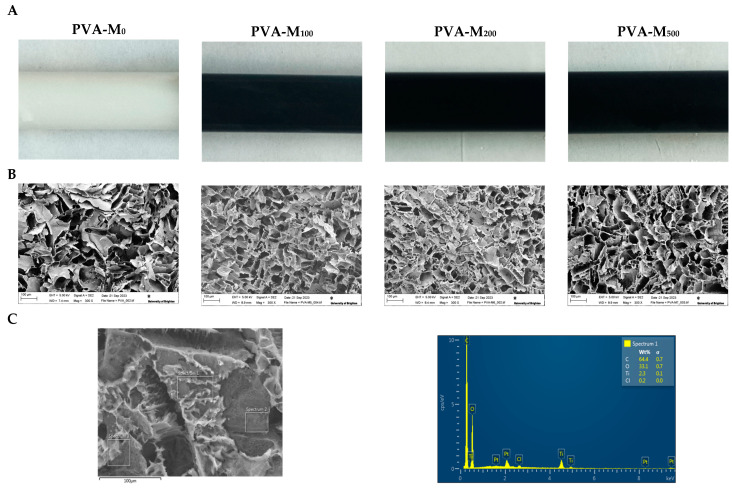
(**A**). Images of as-prepared MXene-containing PVA cryogels. The final concentration of MXene in the PVA solution was 0, 100, 200, or 500 μg/mL. The samples were labeled as PVA-M_0_, PVA-M_100_, PVA-M_200_, and PVA-M_500_. (**B**). SEM images of the cross-sections of MXene-containing PVA cryogels. (**C**). SEM-EDS spectrum for PVA-M_200_ cryogels, showing its constituent elements and the signature elements such as C, O, and Ti. EDS scan area and weight percent (wt.%) of the constituent elements are highlighted.

Figure 1A illustrates the as-prepared cryogels in their cylindrical form (~10 mm diameter). MXene appeared uniformly dispersed throughout the material indicating the presence of a uniform MXene/PVA mixture solution during the gel preparation process. MXene added at concentrations higher than 500 µg/mL was excessive; it was not entirely entrapped in the cryogel and could be washed out of the material, forming undesired aggregates.

According to SEM analysis of the freeze-dried PVA/MXene cryogels, all the samples exhibited a three-dimensional, highly porous structure with large, interconnected pores measuring up to dozens of micrometers in size (Figure 1B). MXene particles were shown to be located in the inner pore walls as there was no visible appearance of plate-like structures on the walls’ surfaces or inside the pores (Figure 1B). The presence of a Ti peak in the EDX spectrum indicates the incorporation of MXene within the polymer walls (Figure 1C and Figure S2).

**Figure 2:**
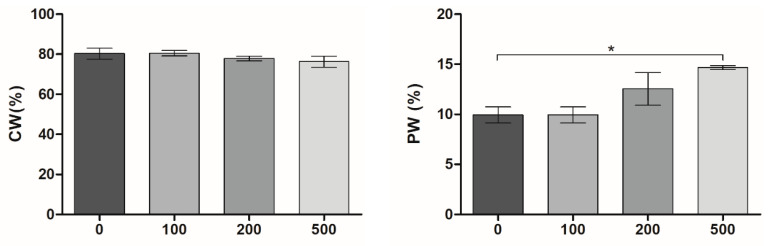
Swelling properties of MXene-containing PVA cryogels. The mass content of capillary water (CW) and polymer-bound water (PW) is shown (mean ± SD, *n* = 3, * *p* < 0.05).

MXene-containing cryogels maintained a significant predominance of capillary water (CW) contained in the pores, over polymer-bound water (PW) retained in the walls (Figure 2), which is a typical characteristic of cryogels [40]. The overall water content in the different cryogel samples was about 90%. The introduction of MXenes had no significant effect on CW. CW can be attributed to the estimation of macropore volume; therefore, its narrow variation between samples would indicate a conservation of the overall macroporous structure with all the MXene concentrations used, whereas the slight increase of PW in proportion to particle concentrations indicates an increased swelling and enlargement of the polymer walls where MXene particles are entrapped [40].

**Figure 3:**
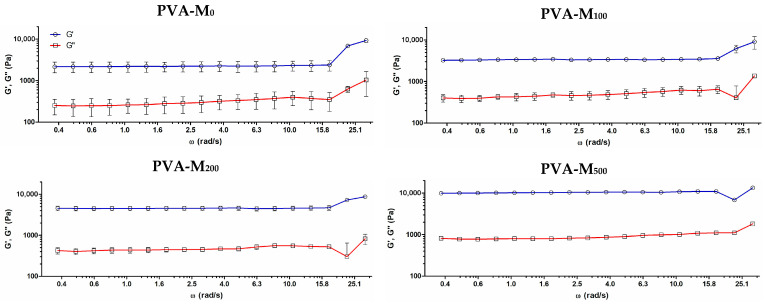
Frequency sweep analysis of MXene-containing PVA cryogels. The measurement of frequency dependence of storage (G′) and loss (G″) modulus was performed within the linear viscoelastic region (LVR) at γ = 0.1% strain deformation.

To investigate the effect of MXene content on the mechanical behavior of the cryogels, rotational rheometry analysis was conducted (Figure 3). The samples of all compositions were characterized by a low damping factor (tan δ << 1); the storage (elastic) modulus (G′) was significantly higher than the loss (viscous) modulus (G″), demonstrating an elastic solid behavior of the materials. The formation of a stable well-structured hydrogel network was confirmed by the independence of G′ and G′′ from increased angular frequency in the studied range (up to ω = 17.5 rad/s, Figure 3) [41,42].

The incorporated MXenes increased the G′/G″ ratio 1.5-fold from 8.4 (PVA-M_0_) to 12.7 (PVA-M_500_), highlighting the contribution of this component to the elasticity and mechanical strength of the formed cryogels. Likewise, according to dynamic mechanical analysis (Figure S3), the compression modulus of the PVA-M_500_ (225.7 ± 3.9 kPa) was ~1.62-times higher than that of MXene-free cryogel (138.8 ± 5.4 kPa), approaching the value reported using a physical surrogate model for human spinal cord tissue in compression (0.2 MPa), also approximating in vivo animal spinal cord properties [43]. The sufficient stiffness of the cryogels must provide them with the mechanical stability to maintain architectural integrity when exposed to deformations [44]. This could be particularly important for in vivo implantation and the design of microchannel conduits to guide axon growth in spinal cord repair [45], since low stiffness may impair the material’s integration with the surrounding tissues and lead to pore collapse [46].

### 2.2. Cell Behavior

#### 2.2.1. Cell Proliferation

To assess the biological effects of the PVA/MXene materials, PC-12 rat pheochromocytoma cells were used as an established model of neural cells [47,48]. The cells were seeded onto the surface of the formed cryogels and allowed to grow for 72 h followed by the assessment of their viability using the MTS proliferation assay.

**Figure 4:**
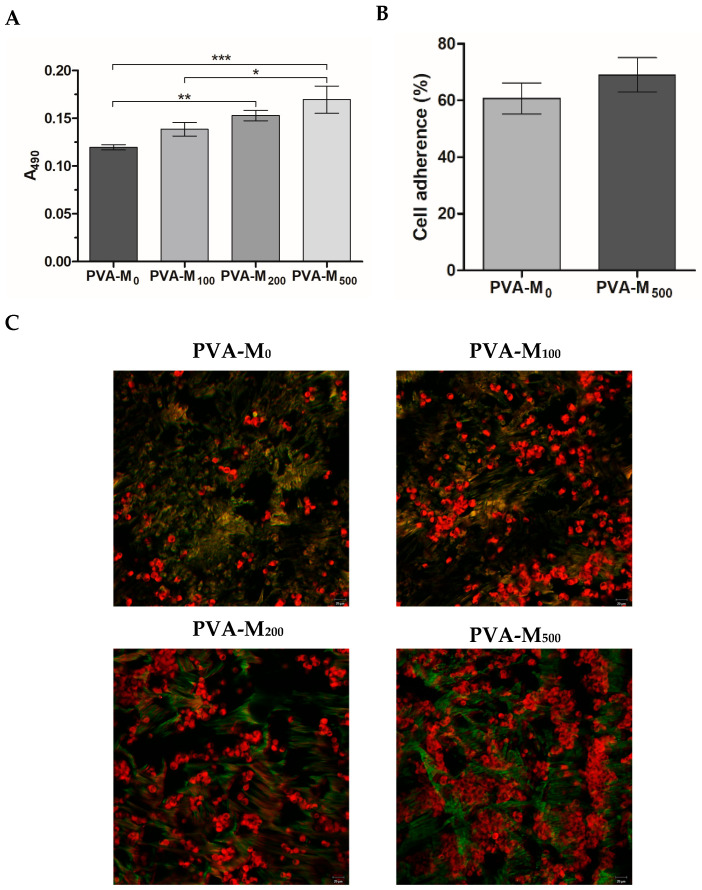
(**A**). Proliferation of PC-12 on MXene-containing cryogels determined by MTS assay on day 3 post-seeding (mean ± SD, *n* = 3, * *p* < 0.05, ** *p* < 0.01, and *** *p* < 0.001). MXene concentrations (µg/mL) are shown. (**B**). Cell adherence on PVA/MXene cryogels after 4 h of incubation (% relative to the total number of seeded cells). (**C**). LSCM of MXene-containing cryogels with PC-12 live cells (red) stained with CellTracker™ Red CMTPX dye on day 3 post-seeding. The cryogels are shown in green due to autofluorescence upon argon laser excitation (514 nm).

In comparison to the MXene suspension added to the culture medium (72 h), which had an IC_50_ value of 199 ± 15 µg/mL, the MXenes embedded in the cryogels did not inhibit cell growth within the studied concentration range. On the contrary, these particles were found to stimulate cell proliferation by 15–44% (Figure 4A). The incorporation of MXenes into the cryogel prevents their immediate release into the medium, which helps reduce their cytotoxicity and allows the cells to grow better in their presence. The lack of cytotoxicity and increase in cell proliferation according to MTS in a time-dependent manner up to 7 days of culture in PVA-M_500_ demonstrates the composite material’s cytocompatibility (Figure S4). Despite the great promise shown by MXenes in biomedical applications, current studies mainly focus on short-term biocompatibility, with insufficient data on long-term toxicity, particularly regarding neurodevelopmental and reproductive effects [18,49]. Comprehensive studies on cytotoxicity mechanisms, immunogenicity, and biodistribution are essential to enhance MXene safety and support their clinical translation.

PC-12 cells showed slightly improved adherence on MXene-modified gels (PVA-M_500_) (Figure 4B), presumably due to increased surface roughness. This result indicates that increased cell number can be partially attributed to enhanced primary adhesion but that it is also associated with the ability of the introduced MXene particles to support cell growth and proliferation. The specific mechanism by which MXenes stimulate this process remains unclear and requires further research; it could presumably be related to their neuroprotective activity and/or their interaction with the cells to modulate ion flux across their membranes and upregulate gene expression to promote cell growth [50,51]. Earlier, MXene-containing Matrigel-based hydrogels showed no cytotoxicity towards seeded neural stem cells (NSCs) for an MXene concentration of 500 μg/mL, at which the expression of proliferation-related genes (Ki67, PCNA and MCM2) was significantly enhanced [29].

The laser scanning confocal microscopy (LSCM) analysis of live cells stained with Cell Tracker showed an increased density of viable cells grown on MXene-containing cryogel compared with MXene-free material (Figure 4C). This further confirms that the corresponding MTS signals (Figure 4A) primarily reflected the cell density in the matrix rather than changes in metabolic activity.

The interconnected macroporous structure of MXene/PVA cryogels obviously improves cell infiltration and growth by supporting nutrient supply and gas exchange, which is essential for neural tissue regeneration and regenerative angiogenesis [52,53].

#### 2.2.2. ROS-Modulating Effects of MXene-Containing Cryogels

**Figure 5:**
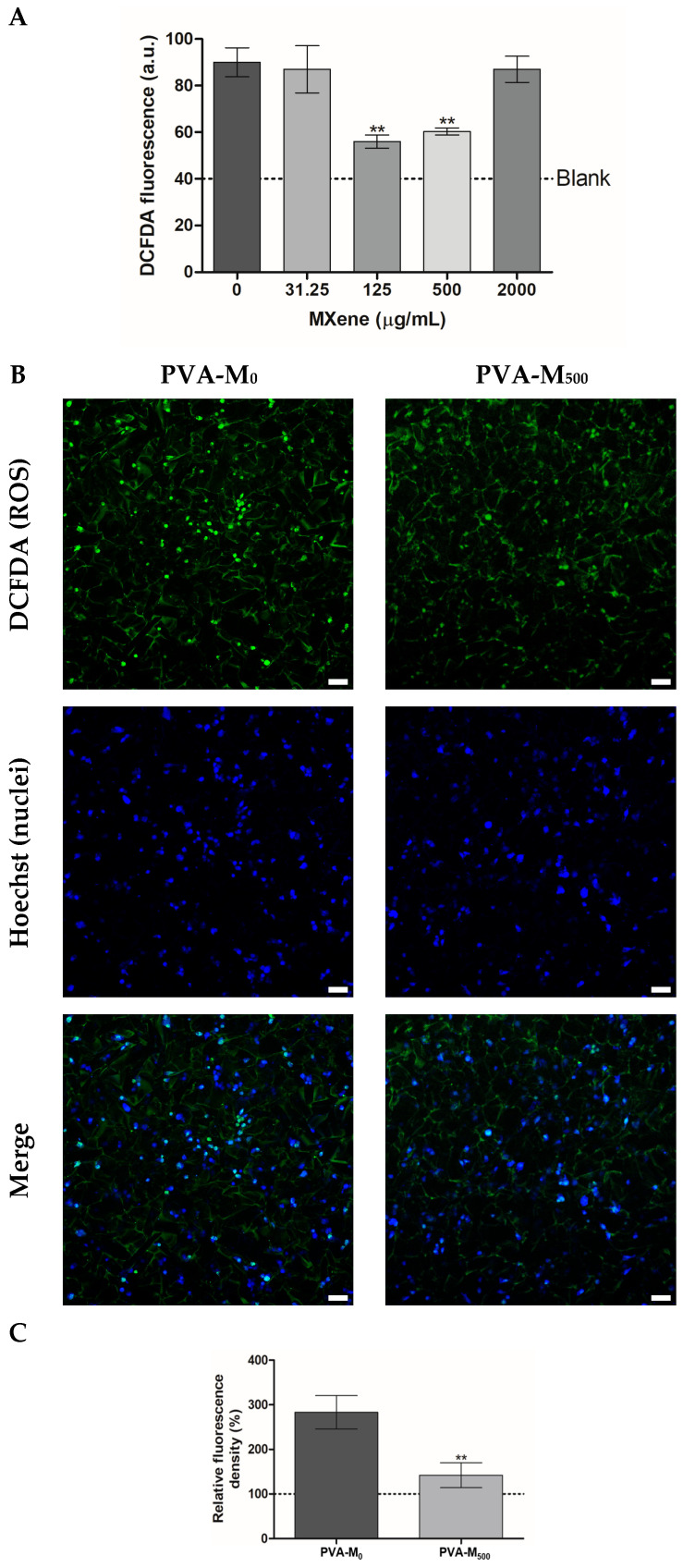
(**A**). Intracellular levels of ROS in PC-12 exposed to 0.5 mM H_2_O_2_ and incubated with MXene-supplemented medium (4 h) (** *p* < 0.01) according to DCFDA fluorescence (λex/λem = 490/526 nm). (**B**). LSCM of MXene/PVA cryogel matrices with live cells co-stained with DCFDA/Hoechst 24 h post-seeding. Scale bar is 20 µm. (**C**). Relative DCFDA fluorescence intensity vs. control value of untreated cells (100%, dotted line) (** *p* < 0.01).

Intracellular levels of reactive oxygen species (ROS), reflecting the oxidative state of the cells, were detected in living PC-12 cells grown on microplate and cryogel matrices. The cells were subjected to hydrogen peroxide (H_2_O_2_)-induced oxidative stress to examine the cytoprotective ability of MXenes [54,55]. According to DCFDA fluorescence, MXenes supplemented to the culture medium of PC-12 at concentrations of 125 and 500 µg/mL considerably decreased the intracellular ROS levels (Figure 5A), highlighting a potential antioxidant neuroprotective role of these particles. The effects of MXene incorporated in the cryogels were additionally confirmed using LSCM analysis showing PC-12 cells stained with DCFDA 24 h post-seeding, where the detected green fluorescence was considerably lower in the presence of MXene (Figure 5B,C).

Recent studies have confirmed the ROS-scavenging property of MXene [56,57,58]. Such a property can be attributed to the exceptional electron transmission ability of MXene [56], along with its demonstrated artificial nonenzymatic antioxidant activity, which scavenges excessive reactive nitrogen species and ROS, maintaining cellular redox homeostasis and alleviating oxidative stress [58]. Various mimetic enzyme activities of MXenes have previously been revealed; they catalyze toxic superoxide (O^2˙−^) into H_2_O_2_ and O_2_ (superoxide dismutase SOD-like activity), generate O_2_ from H_2_O_2_ (catalase CAT-like activity), and employ intracellular glutathione (GSH) to catalyze the conversion of H_2_O_2_ to H_2_O and glutathione disulfide (GSSG) (glutathione peroxidase GPx-like activity) [57]. By removing excess ROS in macrophage cells, 2D Ti_3_C_2_T_x_ MXenes were able to alleviate oxidative stress and induce M1 macrophage transition to a pro-reparative phenotype M2 macrophage thereby reducing inflammation and promoting the proliferation and differentiation of myoblasts and endothelial cells [59].

#### 2.2.3. Neurogenic Differentiation

**Figure 6:**
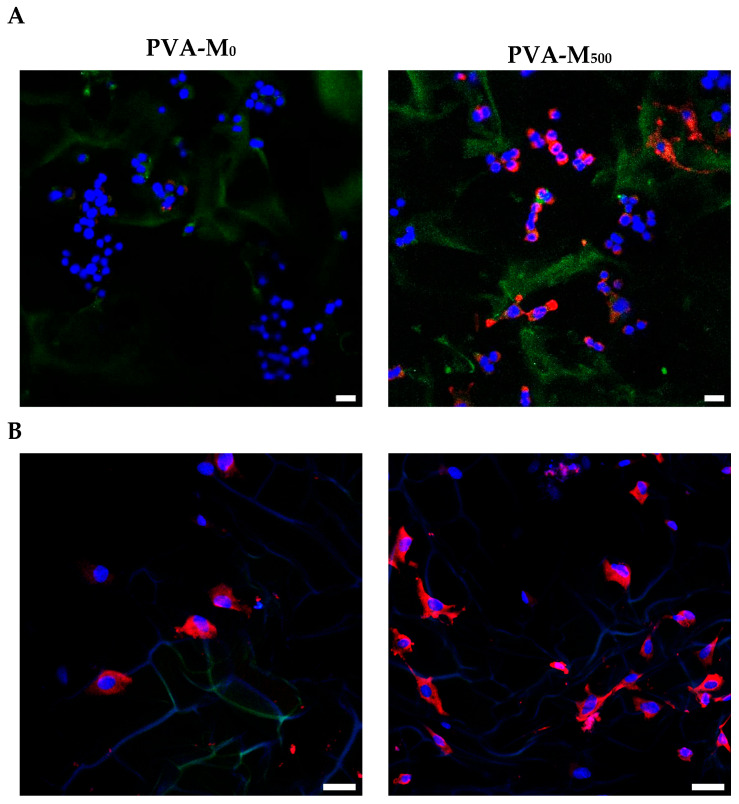
(**A**). Immunofluorescence detection of β-III-tubulin (red) in PC-12 cells on day 5 post-seeding on top surface of MXene-containing PVA cryogels (scale bar is 20 µm). (**B**). PC-12 cells stained with phalloidin CruzFluor™ 647 conjugate for F-actin visualization (red) (scale bar is 50 µm). Corresponding DAPI-stained nuclei are shown (blue).

The ability of MXenes per se to induce a differentiation-like process was assessed in the absence of added growth factors and electrical stimulation. According to immunocytochemistry analysis, PC-12 cells cultured in MXene-containing cryogels had significantly higher expression of the neural specific marker, β-III-tubulin (Tuj1) (Figure 6A). The phalloidin CruzFluor™ 647-stained cells showed altered morphology with reorganization of the actin cytoskeleton in the presence of MXene. A number of elongated neurite-bearing cells were detected, indicating the induction of a neuritogenesis-like process by the introduced particles (Figure 6B). Compared with the control (PVA-M_0_), the cells on PVA-M_500_ had improved spreading with an approximately 1.7-fold higher cell area. The detected mean cell area was, respectively, 207 ± 25 and 352 ± 38 μm^2^.

MXene particles added to a suspension of NSCs were reported to influence intercellular adhesion promoting the formation of 3D spheroids and to induce the latter’s neuronal differentiation [60]. Likewise, Guo et al. showed that Ti_3_C_2_T_x_ MXene film significantly enhanced NSC differentiation towards neurons as demonstrated by Tuj1 and MAP2 upregulation and the increase in length and number of neurites [26]. The combination of decellularized umbilical cord with MXene-containing methacrylate gelatin hydrogel-based nerve conduit synergistically promoted PC-12 neuronal-like differentiation and enhanced β-III tubulin expression and neurite extension from the dorsal root ganglion under physiological conditions [61].

Altogether the obtained results demonstrated the cell-supporting properties of the MXene-containing composite cryogel scaffold along with its ability to exert neuroprotective effects and promote neurodifferentiation. This paves the way for further study of additional properties such as electroconductivity and the application of this material in combination with electrostimulation strategy which is expected to improve its regenerative effects in vitro and in vivo [62,63]. For instance, under excitation of the rotating magnetic field, the conductive MXene/PLLA-based conduit generated an electric current that effectively triggered the PC-12 cell proliferation and neurite outgrowth, as well as the expression of differentiation-related mRNA such as Nestin, MAP2, and Tuj1 [64].

## 3. Materials and Methods

### 3.1. Reagents

The polyvinyl alcohol (PVA, MW~31,000–50,000), 4′,6-diamidino-2-phenylindole (DAPI), phenazine methosulfate (PMS), Triton X-100, and 2′,7′-dichlorofluorescin diacetate (DCFDA) were purchased from Sigma-Aldrich (St. Louis, MO, USA). The Monochlorobimane (MCB), Alamar blue (resazurin), and CellTracker™ Red CMTPX dye were purchased from ThermoFisher Scientific (Waltham, MA, USA). The glutaraldehyde, hydrochloric acid (HCl), and ethanolamine were purchased from TCI (Portland, OR, USA). The 3-(4,5-dimethylthiazol-2-yl)-5-(3-carboxymethoxyphenyl)-2-(4-sulfophenyl)-2H-tetrazolium (MTS reagent) was purchased from Promega (Madison, WI, USA). Ultrapure water (18.2 MΩ·cm, Milli-QAdvantageA10, Merck Millipore, Darmstadt, Germany) was used to prepare aqueous solutions and buffers.

### 3.2. Ti_3_AlC_2_ MAX Synthesis

The MAX precursor was synthesized as detailed previously [35]. TiC (<2 µm, 99.5%), Ti (−325 mesh, 99.5%), and Al (−325 mesh, 99.5%) obtained from Alfa Aesar (Ward Hill, MA, USA) were used. To produce Ti_3_AlC_2_, a 2:1:1 atomic ratio of TiC:Ti:Al (50 g total) was mixed. The powder mixture was then mixed in a 2:1 ball:powder ratio with 5 mm alumina balls. The mixtures were ball milled at 60 rpm for 24 h prior to high-temperature annealing. The high-temperature annealing step was conducted in a Carbolite furnace, with a heating and cooling rate of 3 °C and 200 cm^3^ min^−1^ flow of ultrahigh purity Ar (99.999%). The mixture was heated to 1400 °C for 2 h. After cooling, the porous compacts were milled using a TiN-coated milling bit and sieved through a 400-mesh sieve, producing powders with a particle size < 38 µm.

### 3.3. Ti_3_C_2_T_x_ MXene Synthesis

The MXene was synthesized according to [36]. One gram of Ti_3_AlC_2_ MAX powder was slowly added to a 20 mL mixture of concentrated HF (48 wt%), concentrated HCl (36 wt%), and DI water, with a volumetric ratio of 1:6:3. The MAX phase was etched at 35 °C for 24 h. After the reaction was completed, the etching product was washed with DI water via multiple centrifugation steps (3500 rpm, 5 min) until neutral pH. To delaminate the MXene sheets, the Ti_3_C_2_T*_x_* multi-layer product was stirred at 300 rpm in a 50 mL aqueous solution containing 1 g of LiCl at 35 °C for 18 h. Then, LiCl was removed from the solution by two centrifugation steps (3500 rpm, 5 min), discarding the clear supernatant. After that, the Ti_3_C_2_T*_x_* MXene was completely redispersed in water via shaking and centrifuged for 15 min at 3500 rpm (to precipitate the multi-layer MXene and unreacted MAX particles). Finally, the dark supernatant―containing single-layer MXene sheets―was collected. The SEM images are given in Supplementary Figure S1.

### 3.4. Preparation of MXene-Containing PVA Cryogels

The aqueous solution of PVA (5 wt%) was initially prepared by dissolving the polymer in milli-Q water. MXene-containing PVA cryogels were synthesized using the cryotropic gelation technique [38] after adding MXene to the PVA solution at final concentrations of 100, 200, or 500 μg/mL. The samples were labeled as PVA-M_0_, PVA-M_100_, PVA-M_200_, and PVA-M_500_, respectively. The pH was adjusted to 1–1.2 using HCl (6 M), and the polymers were crosslinked with glutaraldehyde. The mixture was subsequently poured into a glass tube (with an internal diameter of 9 mm), cooled at −12 °C for 4 h in a cooling thermostat, and then kept at −18 °C for 24 h in the freezer. The obtained cryogels were thawed and washed in milli-Q water at room temperature, followed by treatment with ethanolamine solution (0.3 M) to couple the unreacted aldehyde residues.

### 3.5. Scanning Electron Microscopy

For scanning electron microscopy (SEM), the cross-sections of the cryogels’ cylinders were freeze-dried. The SEM analysis was carried out using a Carl Zeiss SIGMA Field Emission Scanning Electron Microscope FEG-SEM; Oberkochen, Germany.

### 3.6. Swelling and Viscoelastic Properties of the Cryogels

The cryogel specimens were sectioned into 3 mm discs and equilibrated with deionized water. The swelling properties of the cryogels were determined by calculating the relative water content in the macropores, known as capillary water (CW), and polymer-bound water (PW) using the following formulas:

CW_%_ = (m_1_ − m_2_)/m_1_ × 100% and PW_%_ = (m_2_ − m_0_)/m_1_ × 100%,

where m_1_, m_2_, and m_0_ represent, respectively, the mass of the fully hydrated, partially hydrated (after removal of the weakly bound water or CW on filter paper), and completely dried (in a thermostat at 90 °C) cryogel materials.

Rheological measurements of the swollen cryogels were carried out on a DHR 2 rheometer (TA Instruments, New Castle, DE, USA) at 37 °C. To determine the linear viscoelastic region (LVR), a strain amplitude sweep test for the cryogels was performed by plotting the storage modulus vs. oscillatory strain γ (%) at an angular frequency ω = 5 rad·s^−1^ (Figure S3). Frequency dependences of the elastic modulus (G′) and loss modulus (G″) were determined using the oscillation mode within the LVR (strain γ = 0.1%).

Dynamic mechanical measurements of the cryogel samples (5 mm thickness) were carried out using the DMA 242 analyzer (NETZCH Instruments, Selb, Germany). Young’s modulus was calculated according to the initial linear region of the stress–strain diagram (E = σ/ε where σ is the stress and ε is the proportional deformation).

### 3.7. Cell Culture and Viability

Rat pheochromocytoma PC-12 cells were cultured aseptically in DMEM supplemented with 10% horse serum (HS), 5% fetal bovine serum (FBS), 2 mM L-glutamine, 100 U/mL penicillin, and 100 μg/mL streptomycin at 37 °C in a humidified air atmosphere with 5% CO_2_.

MXene cytotoxicity was assessed using a preoptimized Alamar blue (resazurin) microplate proliferation assay [54] after culturing the PC-12 cells on a 96-well plate (5 × 10^3^ cells/well) in the presence of MXene (concentration range: 0.5–1000 μg/mL) for 72 h. The cell viability was calculated relative to untreated cells (100% viability).

### 3.8. Cell Seeding and Detection in the Cryogels

Cryogel samples were cut into pieces of 5 mm in height, placed in a 24-well plate, incubated in penicillin (2.5 kU/mL)/streptomycin (2.5 mg/mL) in Hank’s balanced salt solution (HBSS) for 1 h, rinsed, and then equilibrated with full culture medium for an additional 1 h [39]. PC-12 cells were seeded on the top of the cryogels (6.4 × 10^4^ cells/cm^2^) and cultured for 72 h under standard conditions.

#### 3.8.1. Cell Proliferation Analysis

To assess the cell viability/proliferation, the cryogels with grown cells were transferred into new wells and incubated in fresh culture medium supplemented with MTS/PMS reagents for 1 h (in a CO_2_ incubator, 37 °C). The optical absorbance of the generated formazan product was detected at 490 nm on an Infinite M200 PRO microplate analyzer (Tecan, Maennedorf, Switzerland) as a measure of the number of viable cells.

#### 3.8.2. Cell Visualization

The cell-seeded cryogels were stained with CellTracker™ Red CMTPX dye (10 μM, 45 min) in culture medium under growth conditions.

### 3.9. Detection of Intracellular ROS

The effect of MXene dispersion on the intracellular levels of reactive oxygen species (ROS) was investigated. PC-12 cells were seeded on a 96-well plate (2 × 10^4^ cells/well) and allowed to adhere. The cells were exposed to H_2_O_2_ (500 µM) along with MXene at variable concentrations (31.25, 125, 500, and 2000 µg/mL) supplemented to the culture medium. ROS levels were detected following 24 h of treatment by measuring the signals of DCFDA (5 µM, 60 min, λ_ex/em_ = 490/526 nm) using an Infinite M200 PRO microplate analyzer (Tecan).

The MXene/PVA matrices with live cells stained with DCFDA were further visualized and analyzed 24 h post-seeding using an LSCM equipped with a 488 nm argon laser. The cell nuclei were labeled with Hoechst 33342 (5 μg/mL, 15 min). The relative fluorescence of intracellular ROS was calculated using NIH ImageJ 1.48v software and presented as mean ± SD.

### 3.10. Immunocytochemistry

PC-12 cells seeded in the cryogels were cultured in low serum medium (2% HS and 1% FBS) and kept for 5 days under standard culture conditions. Prior to immunocytochemistry, the cryogel matrices with cells were fixed with 4% p-formaldehyde at room temperature (RT) for 1.5 h and gently washed with PBS. After fixation, the samples were incubated in 0.1% Triton X-100 in PBS for 15 min to permeabilize the cell membrane, followed by three washes with PBS. Non-specific binding sites were blocked with 1.5% bovine serum albumin (BSA) for 30 min at RT. Subsequently, the cell-seeded matrices were incubated at 4 °C with APC-conjugated primary antibodies for β-tubulin III at a 1:500 dilution in PBS. Following washing with PBS, the cell nuclei were stained using DAPI. The matrices were visualized by an LSM 780 Zeiss microscope (Carl Zeiss, Jena, Germany). Zeiss Zen black software (2012) was used for acquisition.

Cell morphology was assessed using phalloidin CruzFluor™ 647 conjugate (in 1% BSA, 30 min) for F-actin labeling and cytoskeleton visualization. Cell area was analyzed using ImageJ 1.48v software (National Institute of Health, Bethesda, MD, USA).

### 3.11. Statistical Analysis

Data were presented as mean ± SD. The statistical significance was determined with a Student’s *t* test or one-way analysis of variance (ANOVA), followed by Tukey’s multiple comparison post-test (* *p* < 0.05, ** *p* < 0.01, and *** *p* < 0.001).

## 4. Conclusions

A novel three-dimensional cryogel material with potential uses in neural tissue engineering was prepared by incorporating MXene into PVA cryogel. The incorporated MXenes were shown to contribute to the material stiffness and increased retention of polymer-bound water. The macroporous structure of the obtained composite cryogel provided support for neural cell growth. The MXene/PVA cryogel had good cytocompatibility, where MXene not only lacked toxicity but effectively promoted the proliferation of the cultured neural cells (PC-12) and induced their altered morphology along with the expression of the neural marker (β-III-tubulin). The neuroprotective effect of the MXene component was revealed through the alleviation of oxidative stress and lowered intracellular ROS levels. Overall, these results indicate that MXene-embedded PVA cryogel, due to its macroporous structure, mechanical properties, biocompatibility, and cell-modulating ability, represents a promising material for the development of new strategies in neural tissue engineering.

## Data Availability

The raw data supporting the conclusions of this article will be made available by the authors on request.

## Supplementary Materials

The following supporting information can be downloaded at: https://www.mdpi.com/article/10.3390/molecules30030479/s1. Figure S1: SEM images of MXene nanoparticles; Figure S2: SEM-EDS spectrum for MXene-PVA cryogels, showing their constituent elements and the signature elements such as Ti. EDS scan area and weight percent (wt%) of the constituent elements are shown in the inset; Figure S3: A. Strain amplitude sweep test of the PVA-M_0_ cryogels. The storage G’ modulus is shown as function of strain γ (%) at an angular frequency ω = 5 rad·s^−1^. B. Dynamic mechanical analysis of MXene/PVA cryogels upon gradient increase of compression. Figure S4: Detection of PC-12 cells in PVA-M_500_ cryogel after culturing for 1, 3, 5, and 7 days (MTS, mean ± SD, *n* = 3).

## References

[1] Sjeklocha L., Gatz J.D. (2021). Traumatic Injuries to the Spinal Cord and Peripheral Nervous System. Emerg. Med. Clin. N. Am..

[2] Wang B., Lu C.-F., Liu Z.-Y., Han S., Wei P., Zhang D.-Y., Kou Y.-H., Jiang B.-G. (2022). Chitin scaffold co.bined with autologous small nerve repairs sciatic nerve defects. Neural Regen. Res..

[3] Kornfeld T., Borger A., Radtke C. (2021). Reconstruction of Critical Nerve Defects Using Allogenic Nerve Tissue: A Review of Current Approaches. Int. J. Mol. Sci..

[4] Nan L.-P., Lin Z., Wang F., Jin X.-H., Fang J.-Q., Xu B., Liu S.-H., Zhang F., Wu Z., Zhou Z.-F. (2022). Ti3C2Tx MXene-Coated Electrospun PCL Conduits for Enhancing Neurite Regeneration and Angiogenesis. Front. Bioeng. Biotechnol..

[5] Huang Y.-C., Huang Y.-Y. (2006). Biomaterials and Strategies for Nerve Regeneration. Artif. Organs.

[6] Yergeshov A., Zoughaib M., Dayob K., Kamalov M., Luong D., Zakirova A., Mullin R., Salakhieva D., Abdullin T.I. (2024). Newly Designed PCL-Wrapped Cryogel-Based Conduit Activated with IKVAV Peptide Derivative for Peripheral Nerve Repair. Pharmaceutics.

[7] Kaplan B., Levenberg S. (2022). The Role of Biomaterials in Peripheral Nerve and Spinal Cord Injury: A Review. Int. J. Mol. Sci..

[8] Wang Y., Zhang Y., Zhang Z., Su Y., Wang Z., Dong M., Chen M. (2020). An injectable high-conductive bimaterial scaffold for neural stimulation. Colloids Surf. B Biointerfaces.

[9] Zhang Z., Klausen L.H., Chen M., Dong M. (2018). Electroactive Scaffolds for Neurogenesis and Myogenesis: Graphene-Based Nanomaterials. Small.

[10] Zhang Z., Rouabhia M., Wang Z., Roberge C., Shi G., Roche P., Li J., Dao L.H. (2007). Electrically Conductive Biodegradable Polymer Composite for Nerve Regeneration: Electricity-Stimulated Neurite Outgrowth and Axon Regeneration. Artif. Organs.

[11] Zarrintaj P., Zangene E., Manouchehri S., Amirabad L.M., Baheiraei N., Hadjighasem M.R., Farokhi M., Ganjali M.R., Walker B.W., Saeb M.R. (2020). Conductive biomaterials as nerve conduits: Recent advances and future challenges. Appl. Mater. Today.

[12] Ostrakhovitch E.A., Byers J.C., O’Neil K.D., Semenikhin O.A. (2012). Directed differentiation of embryonic P19 cells and neural stem cells into neural lineage on conducting PEDOT–PEG and ITO glass substrates. Arch. Biochem. Biophys..

[13] Dong C., Qiao F., Hou W., Yang L., Lv Y. (2020). Graphene-based conductive fibrous scaffold boosts sciatic nerve regeneration and functional recovery upon electrical stimulation. Appl. Mater. Today.

[14] Liu X., Kim J.C., Miller A.L., Waletzki B.E., Lu L. (2018). Electrically conductive nanocomposite hydrogels embedded with functionalized carbon nanotubes for spinal cord injury. New J. Chem..

[15] Yadav S., Singh Raman A.P., Meena H., Goswami A.G., Bhawna, Kumar V., Jain P., Kumar G., Sagar M., Rana D.K. (2022). An Update on Graphene Oxide: Applications and Toxicity. ACS Omega.

[16] Prajapati S.K., Malaiya A., Kesharwani P., Soni D., Jain A. (2022). Biomedical applications and toxicities of carbon nanotubes. Drug Chem. Toxicol..

[17] Iravani S., Varma R.S. (2021). MXenes and MXene-based materials for tissue engineering and regenerative medicine: Recent advances. Mater. Adv..

[18] Huang K., Li Z., Lin J., Han G., Huang P. (2018). Two-dimensional transition metal carbides and nitrides (MXenes) for biomedical applications. Chem. Soc. Rev..

[19] Sinha A., Dhanjai, Zhao H., Huang Y., Lu X., Chen J., Jain R. (2018). MXene: An emerging material for sensing and biosensing. TrAC Trends Anal. Chem..

[20] Seidi F., Arabi Shamsabadi A., Dadashi Firouzjaei M., Elliott M., Saeb M.R., Huang Y., Li C., Xiao H., Anasori B. (2023). MXenes Antibacterial Properties and Applications: A Review and Perspective. Small.

[21] Unal M.A., Bayrakdar F., Fusco L., Besbinar O., Shuck C.E., Yalcin S., Erken M.T., Ozkul A., Gurcan C., Panatli O. (2021). 2D MXenes with antiviral and immunomodulatory properties: A pilot study against SARS-CoV-2. Nano Today.

[22] Aslam M., Ahmad T., Manzoor M.H., Laiba, Verpoort F. (2023). MXenes as theranostics: Diagnosis and therapy including in vitro and in vivo applications. Appl. Mater. Today.

[23] Noriega N., Shekhirev M., Shuck C.E., Salvage J., VahidMohammadi A., Dymond M.K., Lacey J., Sandeman S., Gogotsi Y., Patel B.A. (2024). Pristine Ti3C2Tx MXene Enables Flexible and Transparent Electrochemical Sensors. ACS Appl. Mater. Interfaces.

[24] Basara G., Saeidi-Javash M., Ren X., Bahcecioglu G., Wyatt B.C., Anasori B., Zhang Y., Zorlutuna P. (2022). Electrically conductive 3D printed Ti3C2Tx MXene-PEG composite constructs for cardiac tissue engineering. Acta Biomater..

[25] Zhong Y., Huang S., Feng Z., Fu Y., Mo A. (2022). Recent advances and trends in the applications of MXene nanomaterials for tissue engineering and regeneration. J. Biomed. Mater. Res. Part A.

[26] Guo R., Xiao M., Zhao W., Zhou S., Hu Y., Liao M., Wang S., Yang X., Chai R., Tang M. (2022). 2D Ti3C2TxMXene couples electrical stimulation to promote proliferation and neural differentiation of neural stem cells. Acta Biomater..

[27] Li Y., Hu Y., Wei H., Cao W., Qi Y., Zhou S., Zhang P., Li H., Li G.-L., Chai R. (2022). Two-dimensional Ti3C2Tx MXene promotes electrophysiological maturation of neural circuits. J. Nanobiotechnol..

[28] Wu W., Ge H., Zhang L., Lei X., Yang Y., Fu Y., Feng H. (2020). Evaluating the Cytotoxicity of Ti_3_C_2_ MXene to Neural Stem Cells. Chem. Res. Toxicol..

[29] Wei H., Gu Y., Li A., Song P., Liu D., Sun F., Ma X., Qian X. (2024). Conductive 3D Ti3C2Tx MXene-Matrigel hydrogels promote proliferation and neuronal differentiation of neural stem cells. Colloids Surf. B Biointerfaces.

[30] Liao M., Hu Y., Zhang Y., Wang K., Fang Q., Qi Y., Shen Y., Cheng H., Fu X., Tang M. (2022). 3D Ti3C2Tx MXene–Matrigel with Electroacoustic Stimulation to Promote the Growth of Spiral Ganglion Neurons. ACS Nano.

[31] Yu Q., Jin S., Wang S., Xiao H., Zhao Y. (2023). Injectable, adhesive, self-healing and conductive hydrogels based on MXene nanosheets for spinal cord injury repair. Chem. Eng. J..

[32] Cai J., Zhang H., Hu Y., Huang Z., Wang Y., Xia Y., Chen X., Guo J., Cheng H., Xia L. (2022). GelMA-MXene hydrogel nerve conduits with microgrooves for spinal cord injury repair. J. Nanobiotechnol..

[33] Chircov C., Ioniță D.-A., Sîrmon A.-M., Neacșu I.A., Ficai A., Gunduz O., Ustundag C.B., Sengor M. (2023). Chapter 3—Natural, synthetic, and hybrid and composite biomaterials for neural tissue engineering. Biomaterials for Neural Tissue Engineering.

[34] Hixon K.R., Lu T., Sell S.A. (2017). A comprehensive review of cryogels and their roles in tissue engineering applications. Acta Biomater..

[35] Mathis T.S., Maleski K., Goad A., Sarycheva A., Anayee M., Foucher A.C., Hantanasirisakul K., Shuck C.E., Stach E.A., Gogotsi Y. (2021). Modified MAX Phase Synthesis for Environmentally Stable and Highly Conductive Ti3C2 MXene. ACS Nano.

[36] Downes M., Shuck C.E., McBride B., Busa J., Gogotsi Y. (2024). Comprehensive synthesis of Ti3C2Tx from MAX phase to MXene. Nat. Protoc..

[37] Shekhirev M., Shuck C.E., Sarycheva A., Gogotsi Y. (2021). Characterization of MXenes at every step, from their precursors to single flakes and assembled films. Prog. Mater. Sci..

[38] Zheng Y., Gun’ko V.M., Howell C.A., Sandeman S.R., Phillips G.J., Kozynchenko O.P., Tennison S.R., Ivanov A.E., Mikhalovsky S.V. (2012). Composites with Macroporous Poly(vinyl alcohol) Cryogels with Attached Activated Carbon Microparticles with Controlled Accessibility of a Surface. ACS Appl. Mater. Interfaces.

[39] Luong T.D., Zoughaib M., Garifullin R., Kuznetsova S., Guler M.O., Abdullin T.I. (2020). In Situ functionalization of Poly(hydroxyethyl methacrylate) Cryogels with Oligopeptides via β-Cyclodextrin–Adamantane Complexation for Studying Cell-Instructive Peptide Environment. ACS Appl. Bio Mater..

[40] Zoughaib M., Dayob K., Avdokushina S., Kamalov M.I., Salakhieva D.V., Savina I.N., Lavrov I.A., Abdullin T.I. (2023). Oligo (Poly (Ethylene Glycol) Fumarate)-Based Multicomponent Cryogels for Neural Tissue Replacement. Gels.

[41] Li W., Huang A., Zhong Y., Huang L., Yang J., Zhou C., Zhou L., Zhang Y., Fu G. (2020). Laminin-modified gellan gum hydrogels loaded with the nerve growth factor to enhance the proliferation and differentiation of neuronal stem cells. RSC Adv..

[42] dos Santos J.F., Couceiro R., Concheiro A., Torres-Labandeira J.J., Alvarez-Lorenzo C. (2008). Poly(hydroxyethyl methacrylate-co-methacrylated-beta-cyclodextrin) hydrogels: Synthesis, cytocompatibility, mechanical properties and drug loading/release properties. Acta Biomater..

[43] Kroeker S.G., Morley P.L., Jones C.F., Bilston L.E., Cripton P.A. (2009). The development of an improved physical surrogate model of the human spinal cord—Tension and transverse compression. J. Biomech..

[44] Eigel D., Werner C., Newland B. (2021). Cryogel biomaterials for neuroscience applications. Neurochem. Int..

[45] Shahriari D., Koffler J.Y., Tuszynski M.H., Campana W.M., Sakamoto J.S. (2017). Hierarchically Ordered Porous and High-Volume Polycaprolactone Microchannel Scaffolds Enhanced Axon Growth in Transected Spinal Cords. Tissue Eng. Part A.

[46] Kubinová Š., Horák D., Hejčl A., Plichta Z., Kotek J., Proks V., Forostyak S., Syková E. (2015). SIKVAV-modified highly superporous PHEMA scaffolds with oriented pores for spinal cord injury repair. J. Tissue Eng. Regen. Med..

[47] Martín-López E., Nieto-Díaz M., Nieto-Sampedro M. (2012). Differential Adhesiveness and Neurite-promoting Activity for Neural Cells of Chitosan, Gelatin, and Poly-l-Lysine Films. J. Biomater. Appl..

[48] Dayob K., Zengin A., Garifullin R., Guler M.O., Abdullin T.I., Yergeshov A., Salakhieva D.V., Cong H.H., Zoughaib M. (2023). Metal-Chelating Self-Assembling Peptide Nanofiber Scaffolds for Modulation of Neuronal Cell Behavior. Micromachines.

[49] Wu J., Yu Y., Su G. (2022). Safety Assessment of 2D MXenes: In Vitro and In Vivo. Nanomaterials.

[50] Cui D., Kong N., Ding L., Guo Y., Yang W., Yan F. (2021). Ultrathin 2D Titanium Carbide MXene (Ti3C2T) Nanoflakes Activate WNT/HIF-1α-Mediated Metabolism Reprogramming for Periodontal Regeneration. Adv. Healthc. Mater..

[51] Song R., Xie H., Liu G. (2024). Advances of MXene-based hydrogels for chronic wound healing. Chin. Chem. Lett..

[52] Jurga M., Dainiak M.B., Sarnowska A., Jablonska A., Tripathi A., Plieva F.M., Savina I.N., Strojek L., Jungvid H., Kumar A. (2011). The performance of laminin-containing cryogel scaffolds in neural tissue regeneration. Biomaterials.

[53] Yergeshov A.A., Zoughaib M., Ishkaeva R.A., Savina I.N., Abdullin T.I. (2022). Regenerative Activities of ROS-Modulating Trace Metals in Subcutaneously Implanted Biodegradable Cryogel. Gels.

[54] Nikolaeva V., Kamalov M., Abdullin T.I., Salakhieva D., Chasov V., Rogov A., Zoughaib M. (2024). Evaluation of GHK peptide-heparin interactions in multifunctional liposomal covering. J. Liposome Res..

[55] Zhao J., Wang T., Zhu Y., Qin H., Qian J., Wang Q., Zhang P., Liu P., Xiong A., Li N. (2024). Enhanced osteogenic and ROS-scavenging MXene nanosheets incorporated gelatin-based nanocomposite hydrogels for critical-sized calvarial defect repair. Int. J. Biol. Macromol..

[56] Wei C., Tang P., Tang Y., Liu L., Lu X., Yang K., Wang Q., Feng W., Shubhra Q.T.H., Wang Z. (2022). Sponge-Like Macroporous Hydrogel with Antibacterial and ROS Scavenging Capabilities for Diabetic Wound Regeneration. Adv. Healthc. Mater..

[57] Fan L., Lin X., Hong L., Li L., Lin R., Ren T., Tian J., Chen M. (2024). Simultaneous antioxidant and neuroprotective effects of two-dimensional (2D) MXene-loaded isoquercetin for ischemic stroke treatment. J. Mater. Chem. B.

[58] Li Y., Fu R., Duan Z., Zhu C., Fan D. (2022). Artificial Nonenzymatic Antioxidant MXene Nanosheet-Anchored Injectable Hydrogel as a Mild Photothermal-Controlled Oxygen Release Platform for Diabetic Wound Healing. ACS Nano.

[59] Li T., Ma J., Wang W., Lei B. (2023). Bioactive MXene Promoting Angiogenesis and Skeletal Muscle Regeneration through Regulating M2 Polarization and Oxidation Stress. Adv. Healthc. Mater..

[60] Kang Y., Park H., Shim S., Karima G., Lee S., Yang K., Kim H.D. (2025). MXene Nanoparticles: Orchestrating Spherioidogenesis for Targeted Osteogenic and Neurogenic Differentiation. Adv. NanoBiomed Res..

[61] Zhang B., Zhang H., Hu Y., Tian L., Cheng H., Wang Y., Gao X., Cui Q., Zheng S., Feng P. (2024). Decellularized umbilical cord wrapped with conductive hydrogel for peripheral nerve regeneration. Aggregate.

[62] Wu P., Chen P., Xu C., Mu C., Zou X., Yang K., Xu Y., Li X., Li X., Liu Z. (2024). Biodegradable conductive hydrogels generating magnetic-field-driven wireless electrical stimulation enhance the spinal cord injury repair. Nano Energy.

[63] Kim H.-S., Baby T., Lee J.-H., Shin U.S., Kim H.-W. (2024). Biomaterials-enabled electrical stimulation for tissue healing and regeneration. Med-X.

[64] Qi F., Liao R., Wu P., Li H., Zan J., Peng S., Shuai C. (2023). An electrical microenvironment constructed based on electromagnetic induction stimulates neural differentiation. Mater. Chem. Front..

