# Supplementary material for: MXene-Reinforced Composite Cryogel Scaffold for Neural Tissue Repair
Source: Molecules. 2025 Jan 22;30(3):479. doi: 10.3390/molecules30030479 (PMC11820856; doi:10.3390/molecules30030479)
Supplement: Supplementary file 1 [file molecules-30-00479-s001.zip › molecules-3368377-supplementary.pdf]

*Supplementary Materials*

# MXene-Reinforced Composite Cryogel Scaffold for Neural Tissue Repair

**Mohamed Zoughaib [1,2], Svetlana Avdokushina [1,2,3] and Irina N. Savina [3,*]**

1 Institute of Fundamental Medicine and Biology, Kazan (Volga Region) Federal University, 18 Kremlyovskaya St., 420008 Kazan, Russia
2 Scientific and Educational Center of Pharmaceutics, Kazan (Volga Region) Federal University, 18 Kremlyovskaya St., 420008 Kazan, Russia
3 School of Applied Sciences, University of Brighton, Huxley Building, Lewes Road, Brighton BN2 4GJ, UK
* Correspondence: i.n.savina@brighhton.ac.uk

## 1. MXene nanoparticles characterisation

The SEM image of the MXene nanoparticle suspension obtained shows the plate-like morphology of nanoparticles

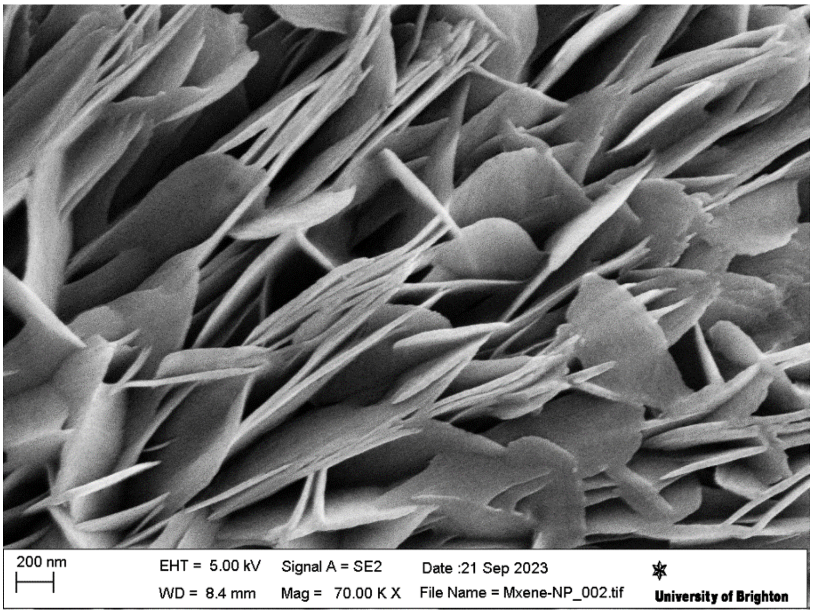


**Figure S1**. SEM images of MXene nanoparticles.

## 2. Analysis of the PVA-$M_{200}$ cryogel wall composition

SEM-EDS spectra of PVA-$M_{200}$ cryogel were taken to analyze the composition of the polymer walls. All three areas analyzed showed the presence of the key elements: C, O, Ti, and Cl. The presence of the Ti peak confirms the incorporation of MXene particles within the polymer wall, with a weight percentage ranging from 2.3% to 2.9%.

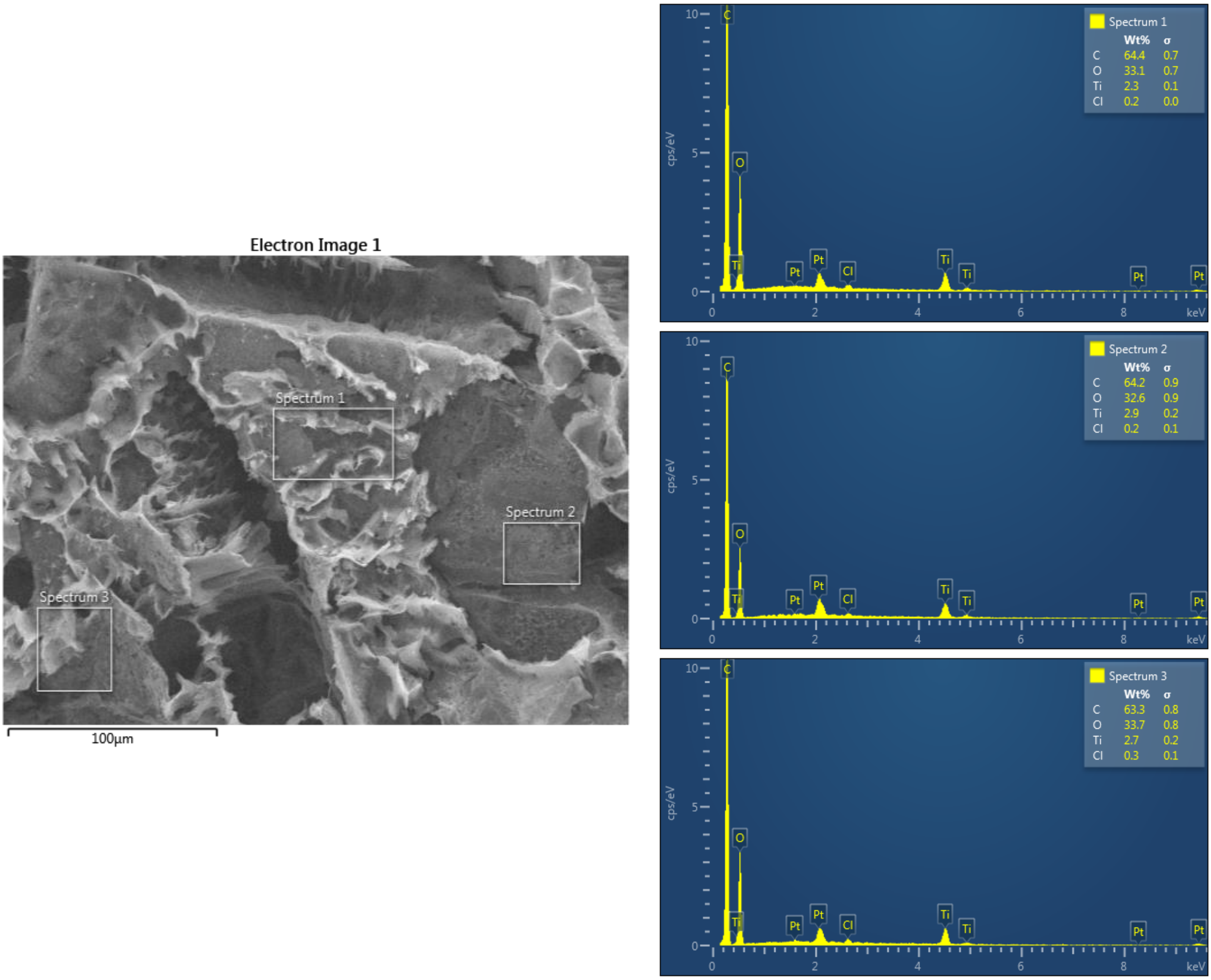


**Figure S2.** SEM-EDS spectrum for MXene-PVA cryogels, showing its constituent elements and the signature elements like Ti; EDS scan area and weight percent (Wt.%) of the constituent elements are shown in the inset. SEM (Carl Zeiss SIGMA Field Emission Scanning Electron Microscope FEG-SEM; Oberkochen, Germany).

### 3. Dynamic mechanical analysis of MXene-functionalized cryogels

A

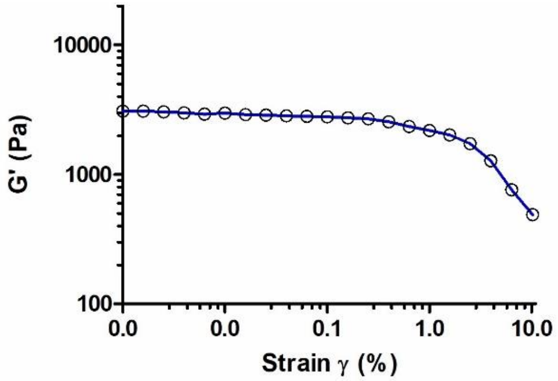


B PVA-$M_0$ PVA-$M_{500}$

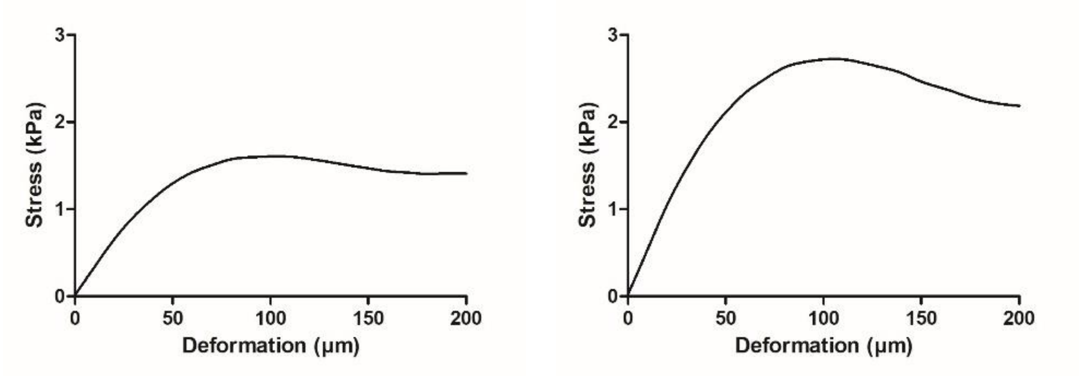


**Figure S3. A.** Strain amplitude sweep test of the PVA-$M_0$ cryogels. The storage G' modulus is shown as function of strain γ (%) at an angular frequency ω = 5 rad.$s^{-1}$. **B.** Dynamic mechanical analysis of MXene/PVA cryogels upon gradient increase of compression.

## 4. Cell proliferation in MXene-functionalized cryogel as a function of culture time

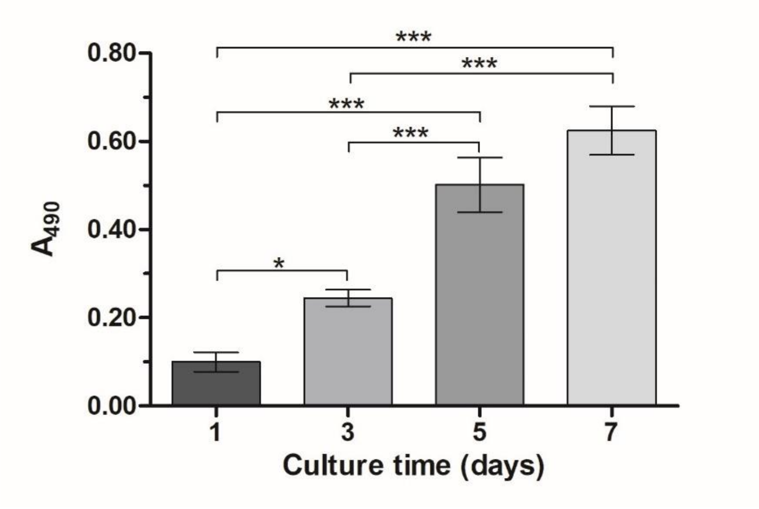


**Figure S4.** Detection of PC-12 cells in PVA-$M_{500}$ cryogel after culturing for 1, 3, 5, and 7 days (MTS, mean ± SD, n = 3).
